# Mesenchymal stem cell as a novel approach to systemic sclerosis; current status and future perspectives

**DOI:** 10.1186/s13619-020-00058-0

**Published:** 2020-12-01

**Authors:** Mina Abedi, Sepideh Alavi-Moghadam, Moloud Payab, Parisa Goodarzi, Fereshteh Mohamadi-jahani, Forough Azam Sayahpour, Bagher Larijani, Babak Arjmand

**Affiliations:** 1 Cell Therapy and Regenerative Medicine Research Center, Endocrinology and Metabolism Molecular-Cellular Sciences Institute, Tehran University of Medical Sciences, Tehran, Iran; 2 Metabolomics and Genomics Research Center, Endocrinology and Metabolism Molecular-Cellular Sciences Institute, Tehran University of Medical Sciences, Tehran, Iran; 3 Brain and Spinal Cord Injury Research Center, Neuroscience Institute, Tehran University of Medical Sciences, Tehran, Iran; 4 Department of Stem Cells and Developmental Biology, Cell Science Research Center, Royan Institute for Stem Cell Biology and Technology, ACECR, Tehran, Iran; 5 Endocrinology and Metabolism Research Center, Endocrinology and Metabolism Clinical Sciences Institute, Tehran University of Medical sciences, Tehran, Iran

**Keywords:** Anti- fibrotic effect, Autoimmune disease, Cell therapy, Immunomodulation, Mesenchymal stem cells, Regenerative medicine, Systemic sclerosis

## Abstract

Systemic sclerosis is a rare chronic autoimmune disease with extensive microvascular injury, damage of endothelial cells, activation of immune responses, and progression of tissue fibrosis in the skin and various internal organs. According to epidemiological data, women’s populations are more susceptible to systemic sclerosis than men. Until now, various therapeutic options are employed to manage the symptoms of the disease. Since stem cell-based treatments have developed as a novel approach to rescue from several autoimmune diseases, it seems that stem cells, especially mesenchymal stem cells as a powerful regenerative tool can also be advantageous for systemic sclerosis treatment via their remarkable properties including immunomodulatory and anti-fibrotic effects. Accordingly, we discuss the contemporary status and future perspectives of mesenchymal stem cell transplantation for systemic sclerosis.

## Background

Systemic sclerosis (SSc) or scleroderma as a rare heterogeneous and chronic autoimmune disease (characterized by extensive microvascular injury, damage of endothelial cells (ECs), activation of immune responses, and progression of tissue fibrosis) can affect the skin and various internal organs including the heart, lungs, kidneys, and gastrointestinal tract (Sobolewski et al. 2019a; Asano 2018; Sticherling 2012; Pattanaik et al. 2015). Of course, cutaneous manifestations (skin thickening and sclerotic skin lesions) are among the main and primary hallmarks of SSc (Krieg and Takehara 2009; Krieg and Takehara 2006). Generally, age, gender, and race as pivotal factors can determine SSc susceptibility. In this respect, females are more susceptible to SSc than males and the most prevalent age of involvement is 30–50 years (Mayes 2003; Nikpour et al. 2010). The subtype of the SSc, the disease stage, and the type of involved organs are crucial elements to determine the best approach for treatment, as well as the most appropriate course of treatment (Sobolewski et al. 2019b; Bissell et al. 2017). Herein, various therapeutic options are offered to control and manage the SSc symptoms. Collectively, mentioned therapeutic options include chemical drugs (prescribed based on the affected organs such as steroid drugs, immune system regulators, and gastrointestinal medications), moisturizers (herbal and/or chemical), phototherapy (especially the use of UVA-1 light to treat skin thickening and lesions), exercise, physical therapy, and occupational therapy (Adnan 2008; Sapadin and Fleischmajer 2002). Moreover, in recent decades, several cell-based therapies and regenerative medicine approaches (as promising and novel alternative options) have studied for SSc and many other chronic diseases (Song et al. 2017; Zakrzewski et al. 2019; Goodarzi et al. 2015; Goodarzi et al. 2014; Payab et al. 2018; Soleimani et al. 2016). In other words, stem cell transplantation has developed as a new way to salvation from different life-threatening autoimmune diseases. Indeed, cell therapy can be effective by preventing the abnormal functioning of self-activated immune cells with the help of safe and effective immune tolerance formed by transplanted cells (Rosa et al. 2007; Resnick et al. 2017; Del Papa et al. 2018; Dazzi et al. 2007; Radbruch and Thiel 2004). Among the various cells, mesenchymal stem cells (MSCs) (obtained from diverse tissues) are mostly known as perfect agents to the regenerative objects to represent cell multi-potency along with anti-inflammatory and powerful regenerating qualities (Larijani et al. 2015; Han et al. 2019; Pittenger et al. 2019; Goodarzi et al. 2018a; Goodarzi et al. 2019a). Furthermore, a broad range of in vitro and in vivo preclinical investigations have shown that autologous and allogeneic MSCs can be employed in a variety of immune-mediated statuses (Zhao et al. 2016; Figueroa et al. 2012). Hence, in the current review, we discuss the contemporary status and future perspectives of MSCs - based therapeutic approaches for systemic sclerosis as an immune-mediated condition.

## A review of scleroderma symptoms and pathophysiology

**Fig. 1:**
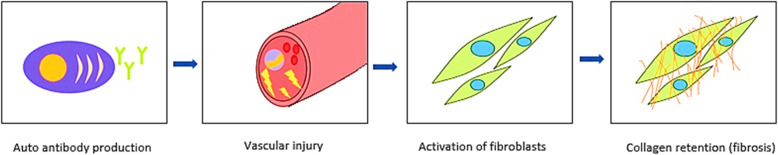
Pathophysiology of Systemic Sclerosis (SSc). In the pathophysiology of SSc first of all, an immune dysregulation is caused that is displayed by auto antibody production. The mechanism during which the dyscrasias is caused, is not completely understood, but it is proposed that genetics and the environmental factors play important roles in the process. Autoantibodies as well as other factors like high levels of reactive oxygen species(ROS) cause vascular injury, the process that leads to the activation of fibroblasts. Activated fibroblasts produce an unusual high amount of collagen (especially type I) as well as other components of extracellular matrix (ECM) that form an extensive amount of fibrosis (Velier et al. 2019)

SSc is an autoimmune disease with a prevalence of 100 to 300 per million depending on the country. Furthermore, it is a disabling disease that affects the quality of life (Maria et al. 2016a; Rozier et al. 2018) by causing symptoms like loss of hand function, pain, and psychological problems. It also reduces life expectancy by causing death from SSc-related complications and raising the incidence of malignancy and cardiovascular problems (Rozier et al. 2018; Elhai et al. 2017; Poudel et al. 2018; Zeineddine et al. 2016). In rapidly progressive forms of the disease, the five-year mortality rate can reach 30–50% depending on the extent of skin, cardiopulmonary, and renal involvement (van Laar and Sullivan 2013; Velier et al. 2019). Vasculopathy)which can make one of the earliest symptoms of the disease(, Raynaud’s Phenomenon (happen as well as digital ulcers), pulmonary arterial hypertension, and telangiectasia (Rozier et al. 2018; Velier et al. 2019; Desbois and Cacoub 2016) are associated with SSc. The main characteristic of the disease is the collagen accumulation in the skin and internal organs, especially the lungs, but the involvement of the heart and digestive system is also reported (Maria et al. 2016a; Rozier et al. 2018; Velier et al. 2019; Chighizola et al. 2011; Okamura et al. 2020). As mentioned, there are three main axes which cause the symptoms: autoimmunity, vascular abnormality, and fibrosis (Asano 2018; Maria et al. 2016a; Rozier et al. 2018; Okamura et al. 2020). Additionally, it has been demonstrated that the environment is involved in the pathophysiology of the disease (i.e., oxidative stress) (Rozier et al. 2018). Different types of antibodies are detected in more than 90% of the patients, including anti-DNA topoisomerase I, anti-RNA polymerase, and anticentromere antibodies (Okamura et al. 2020). These antibodies can cause immune system dysregulation and inflammation. This evidence is supported by the elevated levels of advanced oxidation protein products (AOPPs) in sera and decreased total antioxidant capacity in hypochlorous acid (HOCl)-Induced mouse model of SSc which are a reflection of the inflammation level in the body (Maria et al. 2016a). Collagen retention and following tissue fibrosis can be considered as a key feature of SSc. Therein, fibrosis can form in the skin, lung, or other internal organs. Skin fibrosis can cause symptoms like cutaneous wounds (Sobolewski et al. 2019a; Rubio et al. 2018), degeneration of skin appendages such as hair follicles, sweat glands and cutaneous blood vessels (Wei et al. 2011), microstomia, xerostomia (Sobolewski et al. 2019a). Moreover, perivascular inflammatory cell infiltrates (T lymphocytes and monocytes) as well as increased numbers of alpha-smooth muscle actin (α-SMA)-positive myofibroblasts lead to skin atrophy (Sobolewski et al. 2019a; Wei et al. 2011). On the other hand, the main histological lesion of SSc in the lung is nonspecific interstitial pneumonitis (NSIP) that is a result of interstitial inflammation (in the form of patchy infiltration of the alveolar walls with lymphocytes, plasma cells, macrophages and eosinophils into the alveolar walls and a T helper 2 (Th2) positive bronchoalveolar lavage (BAL)test) along with fibrosis spreading (Wei et al. 2011). Another worse prognosis lesion is usual interstitial pneumonia (UIP) that is characterized by patchy fibrosis (Wei et al. 2011). Air space obliteration and honeycombing can be presented as a result of progressive thickening of the alveolar septa (Wei et al. 2011). Among other internal organs, the esophagus is almost always implicated (Sobolewski et al. 2019a; Wei et al. 2011). Concerning renal implications, vascular lesions are common in contrast to glomerulonephritis (Wei et al. 2011). Interstitial and perivascular fibrosis in cardiac tissue may be silent (Sobolewski et al. 2019a) or presented with diastolic dysfunction (Wei et al. 2011). Among several pro-fibrotic microRNAs (miRs), studies have shown that miR-199-3p is increased in the lung, kidney and liver and induces fibrosis as one of the major symptoms of SSc (Rubio et al. 2018; Cardenas et al. 2013). Indeed, miRs are major regulators of gene expression, the same process exists in pro-fibrotic pathways (Rubio et al. 2018; Bowen et al. 2013). Moreover, it is reported that miR-199-3p impairs cutaneous wound healing responses (Rubio et al. 2018; Chan et al. 2012). Caveolin-1 (CAV-1) is the confirmed target of miR-199-3p and plays an important role in sequestering growth factor receptors and blocking their signaling from the plasma membrane (Rubio et al. 2018; Cardenas et al. 2013). Studies have reported that the CAV-1 level is reduced in fibrotic skin and lung diseases (Rubio et al. 2018; Castello-Cros et al. 2011). On the other hand, the phosphorylation of protein kinase B (PKB) or Akt (involved in cell metabolism, growth, proliferation, and survival) is increased during the fibrogenesis process of SSc (Rubio et al. 2018; Mercer et al. 2016; Zhang et al. 2016). Further, PKB is the other impaired pathway in the pathophysiology of scleroderma that is involved in increased levels of cell apoptosis. Increased level of apoptosis markers (such as caspase-9) reflects this evidence (Tashiro et al. 2015). Altogether, the main pathophysiology of the disease can be summarized in Fig. 1 (Velier et al. 2019).There are two main types of systemic sclerosis based on the extent of collagen deposition: diffuse form and the limited one. There is an extensive amount of fibrosis in the diffuse form of the disease, whereas in the limited form, some local points of fibrosis are detected (Rozier et al. 2018; Okamura et al. 2020; LeROY and Medsger Jr 2001). Generally, the pathophysiology of scleroderma types has not been completely understood to the date (Velier et al. 2019; Okamura et al. 2020). However, it has been believed that immune cells and their secretions play an important role in developing the disease (Okamura et al. 2020). In this context, Th2 cytokines, including interleukin (IL)-4, IL-6, and IL-13 are elevated in the patient’s serum which promote fibrosis (Okamura et al. 2020; Hasegawa et al. 1998). In addition, IL-6 (from B cells) is involved in the disease pathogenesis (Okamura et al. 2020; Le Huu et al. 2012; Matsushita et al. 2018). Collectively, current therapeutic methods for SSc are symptomatic treatments which can’t prevent the disease progression as well as curing the fibrosis lesions (Velier et al. 2019). Up to now, the only treatment that can have profits in the severe form of the disease is autologous hematopoietic stem cell transplantation. However, it can be accompanied by high toxicity, as a side effect (Velier et al. 2019; van Laar et al. 2014; Burt and Farge 2018; Sullivan et al. 2018). Hence, providing an appropriate and novel treatment for SSc seems to be necessary (Rozier et al. 2018; Uji et al. 2015).

## The effects of scleroderma on the properties of patient’s mesenchymal stem cells

Until now, several types of research have been conducted around comparing the MSCs derived from healthy individuals to MSCs from SSc subjects (SSc-MSCs) for assessing the effects of scleroderma on the patient’s MSCs. Although some of them have differed in parts of the outcomes, the majority has indicated the following results:

- In all of the SSc-MSC types, there isn’t a significant difference in phenotype and expression of MSCs specific markers compared to healthy ones (Rozier et al. 2018; Larghero et al. 2008; Scuderi et al. 2013).
- All of the SSc-MSC types are maintained the normal MSCs multi- differentiation ability (Rozier et al. 2018).
- All of the SSc-MSC types are maintained the normal MSCs immune modulator and immune suppressive capability (Rozier et al. 2018; Cipriani et al. 2013a).
- All of the SSc-MSC types are maintained the normal MSCs colony forming unit’s capacity (Rozier et al. 2018).
- All of the SSc-MSC types indicate the increases myofibroblastic differentiation (as a hallmark of scleroderma pathogenesis) strength compared to healthy ones (Guiducci et al. 2011).
- All of the SSc-MSC types are shown the excess levels of type II transforming growth factor beta (TGFβ) receptor (TGFβR II) and following that, promoting the TGFβ signaling pathway and increasing the production of type I collagen target genes compared to healthy ones (Guiducci et al. 2011).
- In SSc- BM- MSCs there is an increasing level of senescence markers compared to healthy ones (Rozier et al. 2018; Guiducci et al. 2011).
- SSc- BM- MSCs can produce more specific markers related to pericytes (as cells that have myofibroblast trans-differentiation tendency) than healthy ones (Cipriani et al. 2013b; Cipriani et al. 2014).
- SSc- AD- MSCs are indicated the reduction of proliferative rate as well as decreases the metabolic, migration and invasion activities compared to healthy ones (Cipriani et al. 2013a; Griffin et al. 2017).

## General characters of mesenchymal stem cells and source of extraction

MSCs are multipotent progenitor cells, which can be extracted from different tissues, including bone marrow (BM), adipose tissue (AD), umbilical cord (UC), placenta (PL), and dental pulp (Maria et al. 2016a; Rozier et al. 2018; Velier et al. 2019; Uji et al. 2015; da Silva et al. 2006). They are known by 3 main characteristics: (1) Plastic adherence, (2) expression of the cell surface markers CD73, CD90, CD105, and lack of expression of the hematopoietic markers CD11b or CD14, CD19 or CD79α, CD34, CD45, human leukocyte antigen-DR (HLA-DR), and (3) capacity of differentiating into adipocytes, chondrocytes, and osteoblasts (Maria et al. 2016a; Rozier et al. 2018; Dominici et al. 2006); moreover, they have functions like supporting hematopoietic stem cells differentiation, cell proliferation as well as anti-fibrotic, antiapoptotic, proangiogenic, anti-bacterial, and anti-inflammatory effects (against the mitogen-induced proliferation of T lymphocytes) (Maria et al. 2016a; Rozier et al. 2018; Maumus et al. 2013). These actions are mostly paracrine which means that MSCs secrete effectors in the extracellular space (Rozier et al. 2018; Velier et al. 2019; Maumus et al. 2013; Abbasi-Malati et al. 2018). However, these effects can happen via cell-cell contact (Rozier et al. 2018; Luz-Crawford et al. 2012). On the other hand, MSCs have been proposed to have pericyte like properties (Rozier et al. 2018). Pericytes are microcirculation cells that their cross-talk with EC, regulates angiogenesis (Rozier et al. 2018). Studies have shown that pericytes have multi-lineage differentiation capacities in vitro and they don’t have this property in vivo (Rozier et al. 2018; Guimarães-Camboa et al. 2017). Hence, it has been concluded that part of MSCs (BM-MSCs) are pericytes acting as multipotent progenitors and express pericyte specific markers, including a-SMA, neuron-glial antigen 2(NG2), regulator of G protein signaling 5 (RGS5), desmin, and platelet-derived growth factor receptors (PDGFb-R) (Rozier et al. 2018; Cipriani et al. 2013b; Cai et al. 2009). MSCs vary in differentiation potential or immunomodulatory capacity based on their source (Rozier et al. 2018; Kern et al. 2006; Keyser et al. 2007). They have pleiotropic activity, so their therapeutic function in different diseases has been evaluated such as rheumatic diseases (Rozier et al. 2018; Ruiz et al. 2016), stroke (Rozier et al. 2018; Toyoshima and Yasuhara 2017), lupus and scleroderma (Rozier et al. 2018; Cras et al. 2015), heart diseases (Rozier et al. 2018; Yu et al. 2017), or bone defects (Rozier et al. 2018; Paduano et al. 2017). In addition, their safety has been proved by hundreds of clinical trials (Rozier et al. 2018; Lalu et al. 2012). In fact, the efficacy of using MSCs in SSc treatment is being assessed by several phase I/II clinical trials (Rozier et al. 2018) of which there is a review in the following parts.

## Mechanism of mesenchymal stem cells in counteracting scleroderma symptoms

**Fig. 2:**
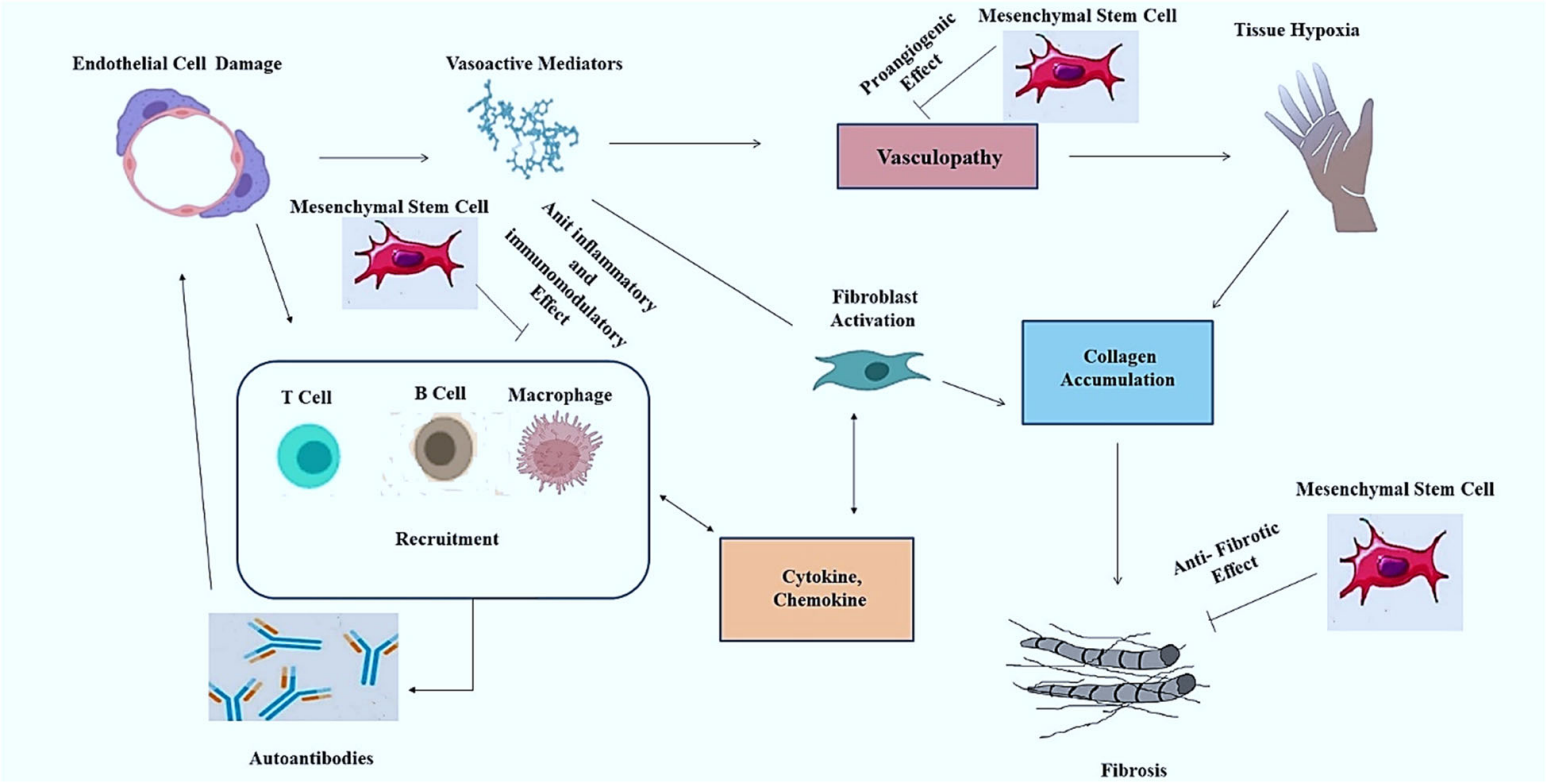
Mesenchymal stem cells (MSCs) Mechanism of Action for Scleroderma Treatment. MSCs can play a role in scleroderma treatment via different mechanism such as an anti-fibrotic effect on collagen accumulation, proangiogenic effect against vasculopathy, and anti-inflammatory and immunomodulatory effects (Peltzer et al. 2018; Lim et al. 2017; Akiyama et al. 2012; Chen et al. 2017a; Maria et al. 2016b)

MSCs are proposed to play important roles in several diseases via different mechanisms of action. Hereupon, various mediators are secreted through the each of these mechanisms (Rozier et al. 2018; Maumus et al. 2013). In this respect, MSCs take part in SSC in 3 main axes (Fig. 2):

- The anti-fibrotic property prevents collagen accumulation in the skin, lung and digestive system (Rozier et al. 2018; Velier et al. 2019). TGFβ1, TGFβR II, collagen type I alpha 1 chain (COL1A1), collagen type III alpha 1 chain (COL3A1), hepatocyte growth factor (HGF), matrix metalloproteinases (MMPs)1, MMP2, MMP9, tissue inhibitor of metalloproteinase 1 (TIMP1), miR-1999-3p, miR-151-5p take part in this axis (Rozier et al. 2018; Rubio et al. 2018; Wang et al. 2016).
- The angiogenic properties, go against widespread vasculopathy by using vascular endothelial growth factor (VEGF), insulin-like growth factor-1 (IGF-1), HGF, platelet-derived growth factor (PDGF), and IL6 (Rozier et al. 2018; Velier et al. 2019).
- The anti-inflammatory property counteracts the dysregulation of the immune system. TGFβ1, IL6, prostaglandin E_2_ (PGE2), HGF, interferon-gamma (IFN-γ), IL-10, IL4, tumour necrosis factor alpha (TNF-α), glucocorticoid-induced leucine zipper (GILZ), indolamine-2,3-dioxygenase (IDO) and inducible NO synthase (iNOS), TNF-α-stimulatedgene-6 (TSG6), interleukin-1 receptor antagonist (IL-1RA), heme oxygenase-1 (HO-1), and TNF receptor 1 (TNFR1) are associated with this mechanism (Rozier et al. 2018; Cras et al. 2015; Maria et al. 2017; Peltzer et al. 2018) (Fig. 2).

### The attenuation of vasculopathy

As mentioned earlier, some of the MSCs have pericyte like properties and enhance vasculogenesis through crosstalks with ECs. Herein, in an experimental study, BM-MSCs (positive for α-SMA) were purified from α-SMA- green fluorescent protein(GFP) transgenic mice in order to study their proliferative and pro-angiogenic characteristics and the conclusion was that α-SMA-GFP positive cells can play a role in neovascularization through special cross-talks with ECs (Cai et al. 2009). When ischemia happens in an organ due to the structural vessel abnormalities during SSc, progenitor cells enter blood circulation from bone marrow or peripheral niches. They adhere to the injured sites and mature into the endothelial and vascular smooth muscle cells (Guiducci et al. 2008). Some of the BM-MSCs are proposed to have the characteristics of endothelial progenitor cells (Guiducci et al. 2008; Chopra et al. 2018). Some studies have shown that although BM-MSCs are negative for vascular endothelial growth factor receptor2(VEGFR2), von Willebrand factor (vWF), and vascular endothelial cadherin factors, growing under endothelial conditions, makes them to express those factors (Chopra et al. 2018; Jiang et al. 2002; Oswald et al. 2004; Reyes et al. 2002). Therefore, it is suggested that BM-MSCs can differentiate into ECs and promote collaterals forming through paracrine mechanisms (Chopra et al. 2018; Kinnaird et al. 2004). Oxidative stress is another mechanism contributing to vasculopathy in SSc. ECs exposure to radical oxygen species causes endothelial aging, morphological changes and impairment of cell–cell adhesion. MSCs can attenuate this process by counteracting the immune dysregulation that happens during the pathophysiology of the disease as well as enhancing antioxidant enzyme production (Guiducci et al. 2008).

### The attenuation of immune dysregulation

Immune dysregulation is another underlying mechanism of the disease that is caused by increased levels of inflammation (reflected by the level of AOPPs in serum) and the reduction of total antioxidant capacity of the body (Maria et al. 2016a). Studies have shown that BM-MSC treatment in HOCL-induced mouse models significantly increases the mRNA expression of the antioxidant enzymes HO-1 and superoxide dismutase 2 (SOD2) in the lung and Sod2 in the skin. Altogether, these effects imply an enhancement in antioxidant capacity of serum in these mice (Maria et al. 2016a).

### The attenuation of fibrosis

Fibrosis spreading in SSc happens during three main mechanisms. All these mechanisms and the effect of MSC treatment on their suppression are discussed in the following part.

- *Reducing the infiltration of immune cells into the skin and lung*

Studies have shown that a polymorphic infiltration of immune cells (including CD3+ T cells and F4/80+ and CD11b + macrophages) as well as myofibroblasts (by α-SMA1 and TGFβ1) plays an important role in the skin and lung fibrosis in bleomycin and HOCl-induced models (Maria et al. 2016a; Okamura et al. 2020; Yoshizaki et al. 2010). However, PAX-51 B cell infiltration wasn’t observed in the organ sections (Maria et al. 2016a; Okamura et al. 2020). A study showed macrophages, neutrophils, and lymphocytes penetration in the lung tissue following intra-tracheal bleomycin injection (Lee et al. 2014). In these studies, AD-MSCs treatment significantly reduced the infiltration of CD4+ and CD8+ T cells, and CD11b + macrophages into the skin compared to the control group (phosphate-buffered saline (PBS)-treated group) (Okamura et al. 2020). The same effect was observed in BM-MSC treatment. More precisely, intra alveolar septa showed almost normal architecture and fewer parenchymal cell infiltration was observed in the lung and skin (Maria et al. 2016a).

- *Reducing mRNA expression of fibrogenic cytokines and collagen*

Producing fibrogenic cytokines (including IL-1b, IL-6, IL-10, IL-13, and TNF-α) is another mechanism that results in the skin and lung fibrosis of SSc in animal models (Maria et al. 2016a; Okamura et al. 2020; Duncan and Berman 1989; Hasegawa et al. 2011; Horikawa et al. 2005; Matsushita et al. 2007; Matsushita et al. 2006). By contrast, IFN-γ (Th1 cytokine) is an antifibrotic cytokine (Okamura et al. 2020; Yamamoto et al. 2000). A study has shown that AD-MSCs treatment in bleomycin-induced mouse models reduces IL-6, IL-13, TNF-α and pro COL1A2 gene mRNA expression in the skin compared to the control group (Okamura et al. 2020). In addition, IFN-γ mRNA levels in the skin of AD-MSCs -treated group were comparable to the control group (Okamura et al. 2020). BM-MSC treatment also resulted in lower levels of IL-1b,IL-6, IL-10 as well as reduced expression levels of COL1, COL3, TGFβ1, and α-SMA genes in the skin and reduced levels of IL-1b, TNF-α, and IL-10 in the lung in HOCL-induced mouse models (Maria et al. 2016a). BM-MSC treatment also reduced expression of transcripts for tissue remodeling parameters, such as tissue inhibitor of metalloproteinases 1 (TIMP-1), HGF, VEGF, and MMP9 in the skin (Maria et al. 2016a). However, the levels of these parameters were a bit different in the lung tissue (Maria et al. 2016a). BM-MSC treatment affected the expression of tissue remodeling parameters in this way: MMp1/ TIMP-1 ratio was increased, along with the reduction of TGFβRII, HGF, MMP9, and VEGFA gene expression (Maria et al. 2016a).

- *Decreasing the frequency of cytokine-producing CD4+ T cells and effector B cells in the spleen in scleroderma models*

Coming to the number of regulatory and cytokine-producing T cells, and effector/regulatory B cells in the spleen, a study has shown that AD-MSCs treatment reduces the number of TNF-α, IL-13 and IL-17-producing CD4+ T cells and IL-6-producing effector B cells in the spleen of bleomycin models. Besides, AD-MSCs can lower the level of IL-13 and IL-17 expression from CD4+ T cells. However, AD-MSCs doesn’t affect the number of T and B regulatory cells (Okamura et al. 2020).

## Animal modeling for experimental studies of scleroderma

In order to be able to have an overall view of the preclinical data accessible about various diseases such as SSc, reviewing the main features of different types of animal models that are most commonly used in this field of study seems to be necessary (Larijani et al. 2019; Goodarzi et al. 2019b). Herein, current review also helps the researchers with the design of relevant animal studies via providing the pros and cons of each model in detail. The SSc preclinical models are divided in two main categories based on the process of the target phenotype developing: the genetic and inducible animal models.

### The genetic animal models

According to studies, scleroderma genetics animal models fall into the following three general categories (Lakos et al. 2004; Artlett 2014; Jimenez and Christner 2002) (Table 1):

1. SSc models which have emerged through spontaneous mutations (lead to raised extracellular matrix deposition) to the genome. This type has demonstrated to be valuable for investigating fibrosis and vasculopathy. Nevertheless, since SSc is not hereditary in humans, the mutations existing in this type cannot be applied to humans.
2. Transgenic models of fibrosis, which are momentous to recognize the part of particular genes related to fibrosis.
3. Knockout models of fibrosis that analyze the specific participation of a gene in fibrosis.

**Table 1:** Scleroderma Genetics Models (Lakos et al. 2004; Jimenez and Christner 2002)

Genetic Model	Category	Specific Features	Site of Fibrosis
Tight skin 1 mouse models(Tsk1/+)	Models with spontaneous mutations	- They are bred as heterozygotes because of lethal homozygous mutation. - They have thickened skin which is tightly joined to the subcutaneous tissue. - The deposition of elastin has risen but there isn’t elasticity in the skin. - They present an emphysema-like pathology in the lung due to the increased elastin. - They have an enlarged heart and skeleton. - There is partial duplication of the fibrillin-1 gene as a mutation on chromosome 2 which seems to suppress raised levels of TGFβ in the extracellular matrix and following stimulating collagen synthesis.	Skin
Tight skin 2 mouse models (Tsk2/+)	Models with spontaneous mutations	- They are generated via mutagenic agent ethylnitrosourea. - Tight skin can be found in the interscapular region. - The mutation is placed on chromosome 1. - They can mimic many characteristics of systemic sclerosis subjects, containing increased deposition of the dermal extracellular matrix, tight skin, and autoantibodies. - They present enhanced transcription percentages of dermal fibroblasts type I and III collagen. - Increased autoimmunity have reported in Tsk2/+ models.	Skin
UCD-200 chickens	Models with spontaneous mutations	- They are identified by infiltration of perivascular lymphocytic, vascular occlusion caused by endothelial injuries, fibrosis of the skin and visceral organs, and spotted antinuclear antibodies. - There is a genetic defect with an autosomal recessive mode of inheritance or multiple loci interactions. - Five genes including which have been reported to have importance in the pathology of systemic sclerosis including (TGFBR1, IGFBP3, EXOC2/IRF4, CCR8 (located on chromosome 2), and SOCS1(located at chromosome 14) have been recognized to present a high association with the disease. - Mutation of COL1A2 gene (located on chromosome 2) may play a role in avian systemic sclerosis.	Skin and Visceral organs
Endothelin-1 mouse models	Transgenic models of fibrosis	- There is increased expression of endothelin-1. - They have endothelial dysfunction. - They develop glomerulosclerosis and interstitial fibrosis.	Kidney and Lung
Fos-related antigen-2 mouse models	Transgenic models of fibrosis	- There is overexpressing the Fos-related antigen-2 (FRA-2) - They show microangiopathy along with progression of skin fibrosis.	Skin and Lung
Type I TGFβ receptor transgenic models	Transgenic models of fibrosis	- There is upregulating of the type I TGFβ receptor in fibroblasts on a Cre-ER transgenic background. - There are increasing levels of collagen deposition in the skin of through the aging.	Skin
Kinase-deficient type II TGFβ receptor transgenic models	Transgenic models of fibrosis	- There is a fibroblast-specific transcriptional enhancer (applied to specifically express a kinase-deficient mutant type II TGFβ receptor which can involve TGFβ) upstream of the COL1A2 collagen gene. - There is the lack of immune activation and autoantibodies.	Skin and Lung
PDGF receptor-α transgenic models of fibrosis	Transgenic models of fibrosis	- There is conditionally expressed PDGF receptor-α activating mutations.	Skin and Internal organs
Caveolin-1 deficient models	Knockout models of fibrosis	- There are raised collagen and fibronectin accumulation along with increased amounts of myofibroblasts. - There is oxidative stress condition.	Skin
Early growth response protein-1 knockout mouse models	Knockout models of fibrosis	- There are reduced amounts of infiltrating inflammatory cells in the skin along with the reduced dermal thickness and expression of collagen.	Skin and Lung
Friend leukemia integration factor-1 conditional knockout mouse models	Knockout models of fibrosis	- There is risen vascular permeability. - They mimic the collagen fibril formation abnormalities in systemic sclerosis.	Skin
Macrophage chemoattractant protein-1 mouse models	Knockout models of fibrosis	- There are reduced fibrotic replies after the injection of basic fibroblast growth factor and connective tissue growth factor into the skin. - There are fewer mast cells, reduction of macrophage recruitment, and fewer CD4+ T-cell amounts.	Skin
Microsomal prostaglandin E2 synthase-1 knockout models	Knockout models of fibrosis	- There are bleomycin-induced fibrosis resistant, α-smooth muscle actin levels reduction, and macrophages numbers reduction.	Skin and Lung
Peroxisome proliferator-activated receptor-gamma deficient models	Knockout models of fibrosis	- There is more extensive skin thickening with bleomycin compared to wild-type. - They can help to determine the function of this protein in collagen synthesis.	Skin
PTEN conditional knockout mouse models	Knockout models of fibrosis	- There are developed thickened skin and collagen deposition along with raised α-smooth muscle actin-bearing fibroblasts numbers, connective tissue growth factor-positive fibroblasts, and proliferating cell nuclear antigen-positive fibroblasts.	Skin
Relaxin knockout mouse models	Knockout models of fibrosis	- There is developed skin collagen deposition over time.	Skin

*Tsk1/+* Tight skin1 mouse, *Tsk2/+* Tight skin2 mouse, *TGFβ* Transforming growth factor beta, *TGFBR1* Transforming Growth Factor Beta Receptor 1, *IGFBP3* Insulin-like growth factor-binding protein 3, *CCR3* C-C chemokine receptor type 3, *SOCS1* Suppressor of cytokine signaling 1, *COL1A2* Collagen Type I Alpha 2 Chain, *FRA-2* Fos-related antigen-2, *ER* Estrogen Receptor, *PDGF* Platelet-derived growth factor

### Inducible animal models

Several animal models are categorized as inducible ones (Asano and Sato 2013; Morin et al. 2015). Every model can mimic particular manifestations of the disease. To the date, mice and rats have been widely used as inducible animal models and are subdivided into several groups based on their target preclinical manifestations such as developing lung or skin fibrosis, mimicking inflammatory phases of the disease, pulmonary hypertension (Storkanova and Tomcik 2017) and presenting exact mechanisms induced by a particular factor. The HOCL-injected model is largely used in preclinical studies of scleroderma and exhibits systemic phenotype of human SSc through developing fibrosis, inflammation and vasculopathy as the main pathophysiological aspects of the disease (Asano and Sato 2013; Morin et al. 2015). A subgroup of models exclusively simulates the pattern of fibrosis extension in pulmonary tissue through fibroblast activation and increasing collagen gene expression (Storkanova and Tomcik 2017). Some models present skin fibrosis through the activation of micro-chimeric fetal cells (Storkanova and Tomcik 2017) and some simulate the inflammatory phases of SSc through immune cell infiltration (Morin et al. 2015; Storkanova and Tomcik 2017; Yamamoto 2010). Several models are designed for pulmonary hypertension studies and the others develop particular processes induced by related factors; for example, the role of anti-topoisomerase immunization, TGFβ, anti-endothelin I receptor type-A and anti-angiotensin II receptor type − 1 antibodies in developing the pathological phenotype can be studied in particular models (Storkanova and Tomcik 2017). Therein, several preclinical models and their different features in addition to advantages and disadvantages are provided in detail in Table 2.

**Table 2:** Preclinical Inducible Models in Studies of Systemic Sclerosis

Target preclinical feature	Animal Models	Observed Characteristics	The Underlying Mechanisms of Model Designing	Advantages	Disadvantages	References
Systemic phenotype of the disease	HOCl-injected mice	-skin, lung and kidney implication -vascular abnormalities -autoantibodies production -↑CD4 + T-cell and B-cell in the spleen	HOCl injection→↑ROS: -↑collagen and α-SMA production in skin - anti-DNA topoisomerase-1 autoantibodies production→ systemic symptoms HOCl injection→↑AOPP→ systemic fibrosis	-presenting the key features of the human disease (in three main aspects of fibrosis, inflammation and vasculopathy) - Presenting the role of ROS and AOPPs in the pathogenesis of SSc	ND	(Rozier et al. 2018; Asano and Sato 2013; Morin et al. 2015)
Skin fibrosis	Vinyl Chloride Injected mice	-skin and spleen fibrosis and cell infiltration -↑IL-4 and IL-13 during a Th2 immune response	vinyl chloride injection→ activation of micro chimeric fetal cells→ cell division→ symptoms presentation	-showing the role of micro chimeric fetal circulating cells and chemical exposure in the pathogenesis of SSc -an easily reproducible model	ND	(Morin et al. 2015; Storkanova and Tomcik 2017; Christner et al. 2000)
Lung fibrosis	Silica-induced lung fibrosis mice	- pulmonary tissue fibrosis	instillation of silica→ macrophage activation→ phagocytosis of silica particles→ pro-fibrotic cytokines(PDGF, TGFβ) production→ lung fibrosis	-mimicking the pulmonary phenotype of long-term exposure to silica dust(as a permanent fibrotic stimuli)	-an expensive animal model -a time consuming process -specialized equipment requirement -lacking the characteristics of UIP	(Storkanova and Tomcik 2017)
	FITC induced lung fibrosis mice	-pulmonary edema, inflammation and fibrosis	FITC usage: - binding to the protein in the lungs→ formation of a new antigen→ antibody formation -↑mononuclear cells and neutrophils infiltration→ acute lung injury -↑CCL12 and CCL2 → ↑CCR2 expressing fibroblasts→ pulmonary fibrosis	-fibrosis detection using green fluorescence. -the phenotype occurs rather fast (during 14–28 days) and continues for at least 6 months.	-lacking the characteristics of UIP	(Storkanova and Tomcik 2017; Chung et al. 2003)
	Radiation-induced lung fibrosis mice	-pulmonary tissue fibrosis	Radiation→ pneumocystis I and II death→ the production of pro-inflammatory and pro-fibrotic cytokines (TGFβ, TNF-α) by macrophages→ fibrosis	-presenting the characteristics of UIP.	-an expensive animal model -a time consuming process	(Storkanova and Tomcik 2017)
Lung fibrosis / Immunogenic/inflammatory features	Bleomycin-Injected mice	-lung and skin fibrosis -↑hydroxyproline -↑type-I collagen -Antinuclear autoantibodies production (anti-Scl-70, anti-U1-RNP, and anti-histone)	Bleomycin injection→↑ROS → endothelial cell damage and ↑adhesion molecules→ leukocytes attraction and fibroblast activation→ fibrosis	-presenting some of the early inflammatory symptoms of the disease. -useful to test the efficacy of anti-fibrotic therapeutics -useful to assess the potential of the pro-inflammatory genes of the patient	-not presenting the typical clinical features and autoantibody patterns of the disease	(Rozier et al. 2018; Morin et al. 2015; Yamamoto 2010)
Immunogenic/inflammatory features	Scl-GVHD mice	-fibrosis formation and chronic inflammation of the skin, lung, and gastrointestinal tract -↑CCL2, CCL5, CCL17, IFN-γ-inducible chemokines, PDGF, CTGF, FGF, EGF, NGF, VEGF and adhesion molecules in the skin	BM and spleen cells transplantation into BALB/cJ (H-2d) mice→ the donor immune cells infiltration+ auto-reactive host T cells escape from thymic negative selection	-demonstrating many clinical and histological similarities to scleroderma	-ND on symptoms of vasculopathy presentation in mice while vasculopathy is one the signs of patients with Scl-GVHD	(Morin et al. 2015; Yamamoto 2010)
Pulmonary atrial hypertension (PAH)	chronic hypoxia+ semaxanib (SU5416)-induced PAH mice	-PAH	hypoxia→ pro-inflammatory cytokines secretion SU5416: -↑growth factors (FGF, PDGF) → endothelial cells proliferation→ PAH -↑shear stress in the artery wall→ angioobliterative PAH	-exhibiting the pathophysiological role of endothelial proliferation of pulmonary artery in PAH	-the hypoxia-induced PAH is slight and reversible	(Storkanova and Tomcik 2017; Nicolls et al. 2012)
	MCTP- induced PAH rats	-PAH	MCTP→ endothelium and smooth muscle cell proliferation and mononuclear inflammatory cells infiltration→ PAH	-presenting the acute process of PAH	-the induced phenotype is easily treatable that is different from PAH in human SSc	(Storkanova and Tomcik 2017; Stenmark et al. 2009)
	SU5416-induced PAH athymic rats	-severe PAH	macrophage, B cell and anti-endothelial antibodies→ pulmonary artery inflammation→ lack of regulatory T cell→ severe PAH	-studying the function of T reg anti-inflammatory cells in counteracting PAH in SSc patients.	ND	(Storkanova and Tomcik 2017; Nicolls et al. 2012)
	ET_A_R and AT_1_R antibodies injected mice	- obliterative vasculopathy of pulmonary vessels -PAH	anti-ET_A_R and anti-AT_1_R injection→↑α-SMA expression and lymphocyte infiltration in perivascular areas→ obliterative vasculopathy, ↑vascular reactivity and vascular remodeling	-useful to assess the roles of ET_A_R and AT_1_R antibodies in the disease pathogenesis	ND	(Morin et al. 2015; Becker et al. 2014)
Phenotypes caused by a particular factor	topoisomerase-1+ CFA injected mice	-skin and lung fibrosis -↑IL-6, TGF-β1, IL-17 and IL-10 -Th2 and Th17 in BAL	topoisomerase-1 + CFA injection→↑Th2/Th17 immune pathway→ skin sclerosis, ILD, and ↑inflammatory cytokines	-mimicking diffuse SSc symptoms -proposing the relationship between responses to topo I and the pathogenesis of the disease. -advantageous for studying the effects of immunosuppressive and anti-inflammatory drugs on SSc.	ND	(Asano and Sato 2013; Morin et al. 2015)
	Angiotensin II-Induced mice	-↑collagen, CTGF, TGFβ and pSmad2 expression -↑hydroxyproline content in skin -↑immune cell infiltration into the skin -↑vascular injury markers(vWF, TSP-1 and MMP-12)	-exogenous angiotensin II: -collagen I receptor stimulation→ skin fibrosis -the dysregulation of endothelial-to-mesenchymal transition→ activated fibroblasts production	-showing the role of the renin-angiotensin pathway in the process of fibrosis formation -advantageous for studying the effects of anti-inflammatory drugs on SSc.	-not mimicking the auto-immune process of the human disease. -blockage of related signaling pathway has little effect on the pathophysiology of the disease	(Asano and Sato 2013; Morin et al. 2015)
	Exogenous TGFβ+ CTGF injected mice	-ECM-rich skin fibrosis -↑macrophages	TGFβ→granulation and fibrotic tissue formation CTGF and bFGF→ sustained ↑collagen I gene expression→ fibrosis maintenance	-presenting sustained fibrosis due to the use of CTGF in combination with TGFβ	ND	(Yamamoto 2017)

*HOCl* Hypochlorous Acid, *CCR* CC chemokine receptor, *FITC* Fuorescein Isothiocyanate, *TNF-α* Tumour Necrosis Factor Alpha, *UIP* Usual Interstitial Pneumonia, *RNP* Ribonucleoprotein, *ROS* Reactive Oxygen Species, *CCL* C-C Chemokine Ligand, *IFN-γ* Interferon-Gamma, *NGF* Nerve Growth Factor, *EGF* Epidermal growth factor, *GVHD* Graft versus host disease, *VEGF* Vascular Endothelial Growth Factor, *FGF* Fibroblast Growth Factor, *PDGF* Platelet-Derived Growth Factor, *SU5416* Semaxanib, *MCTP* Monocrotaline, *PAH* Pulmonary Atrial Hypertension, *ET*_*A*_*R* Anti-endothelin receptor Type-A, *AT*_*1*_*R* Anti-angiotensin Receptor Type − 1, *ILD* Interstitial Lung Disease, *IL* Interleukin, *CFA* Complete Freund’s Adjuvant, *Th* T helper, *pSmad2* phospho-Smad2, *α-SMA* Alpha-Smooth Muscle Actin, *vWF* von Willebrand Factor, *TSP-1* Thrombospondin-1, *MMP* Matrix Metalloproteinases, *SSc* Systemic Sclerosis, *ECM* Extracellular Matrix, *bFGF* Basic Fibroblast Growth Factor, *CTGF* Connective Tissue Growth Factor, *TGFβ* Transforming Growth Factor beta, *ND* No Data

## Animal studies for mesenchymal stem cells - based treatments in scleroderma

In order to have a better overview on the effects of MSC treatment on SSc phenotype, preclinical data from animal studies with a publication date from 2016 till now is reviewed. Bleomycin-induced, HOCl-injected and Tsk1/+ models are three most commonly used animal models in this case. Moreover, among different types of mesenchymal stem cells, there has been a favored toward using BM-MSC in SSc animal experiments. Studies have shown that AD-MSC injection in SSc models counteracts fibrosis expansion in the lung and skin as well as levels of cytokines and gene products involved in fibrosis forming (Okamura et al. 2020; Rubio et al. 2018; Maria et al. 2016b). A better state of wound healing and lower levels of inflammation were also observed in the implicated sites (Rubio et al. 2018; Maria et al. 2016b). The same effects were reported from BM-MSC based experiments (Maria et al. 2016a; Maria et al. 2016b; Lan et al. 2017; Maria et al. 2018) as well as proposing the beneficial effect of HGF in causing BM-MSC to function more efficiently against fibrosis expansion (Cahill et al. 2016). In addition, some studies have reported the potential of thioredoxin 1 (Trx-1) overexpressing BM-MSC in lowering the apoptosis rate (Jiang et al. 2017). BM-MSC injection also decreases osteopenia and bone marrow substitution by adipose tissue in Tsk1/+ SSc models (Chen et al. 2017b). The inhibition of mammalian target of rapamycin (mTOR) pathway has been suggested as the underlying mechanism (Chen et al. 2017b). Beneficial effects of MSC-based therapeutic approaches on animal models of SSc have been provided in more detail in Table 3.

**Table 3:** Some of The MSC-based Animal Studies for Scleroderma

References	Animal models	Origin and dose of MSCs	The site of stem cell injection	Observed outcomes
(Okamura et al. 2020)	Bleomycin intradermic injection/mouse(daily,4 weeks) and Scl-cGVHD(irradiated with 400 cGy twice a day/1 day)	2 × 10^5^ syngeneic AD-MSCs	intravenous	↓dermal thickness ↓skin fibrosis (both models) ↓lung fibrosis (both models) ↓infiltration of immune cells into the skin ↓COL1A2 mRNA expression ↓IL-6 and IL-13 ↓IL-10 and IFN-γ ↓frequency of cytokine producing CD4+ T cells and effector B cells in the spleen
(Maria et al. 2018)	HOCl intradermic injection/mouse (daily, 42 days)	2.5 × 10^5^ syngeneic BM-MSCs	intravenous	↓ skin thickness ↓ IL-1β, TNF-α, IL-6, and IL-10 expression ↓ cellular infiltrates ≠anti-scl70 autoantibody producing ≠skin inflammation ↓myofibroblastic activation (MSC injected on d42) ↑MMP1 (MSC injected on d42) ↓TIMP1 (MSC injected on d42) *all the other results are from MSC injection on d0
(Rubio et al. 2018)	Bleomycin intratracheal/mouse	5 × 105 syngeneic AD-MSCs	intravenous	≠skin fibrosis ≠lung fibrosis ↓ total wound size ↓ expression of miR-199-3p in skin wound tissue and lungs ≠CAV-1 downregulating ≠AKT Phosphorylating ≠inflammatory markers upregulating ≠αv-integrin mRNA upregulating
(Lan et al. 2017)	Bleomycin intratracheal/mouse	2.5 × 10^5^ syngeneic OSM preconditioned BM-MSCs	intratracheal	↑wound healing ↓collagen content ↓ECM synthesis ↓inflammatory mediators ↓lung edema ↓total cells and neutrophils in BAL fluid ↓fibrotic factors in the lung ↓histological changes
(Jiang et al. 2017)	Bleomycin subcutaneous/mouse(daily,21 days)	1 × 10^6^ syngeneic BM-MSCs overexpressing Trx-1	subcutaneous	↓apoptosis ↓ Bax ↓cleaved caspase 3 ↑Bcl-2 ≠Dermal thickening ↓TGFβ, α-SMA, fibronectin and collagen 1 expression in the skin
(Huleihel et al. 2017)	Bleomycin intratracheal/mouse	5 × 105 human BM-MSCs overexpressing let7d	intravenous	↓weight loss ↓CD45 positive cells in the lung ↓collagen transcript levels NC in α-SMA, HMGA-2, N-Cadherin and FSP-1 expression
(Chen et al. 2017b)	Tsk1/+ mouse	1 × 10^5^ allogeneic BM-MSC /10 g bodyweight	intravenous	↑osteoblast and osteoclast numbers in the femurs ↑serum levels of type I collagen cross-linked telopeptide (CTX) and sRANKL ↑bone formation rate ↑CFU-F Improvement of osteogenic differentiation of BM-MSCs in mice ↑Runx2, ALP, and OCN ↓adipocytes in the bone marrow ↓PPARγ and LPL expression
(Maria et al. 2016b)	HOCl intradermic injection/mouse(daily, 42 days)	2.5 10^5^ human BM-MSCs/ AD-MSCs	intravenous	↓rate in skin thickness formation ↓total collagen deposition in skin and lungs ↓COL1, COL3 and α-SMA gene expression ↓infiltration of CD3+ T lymphocytes and F4/80+ macrophages ↑MMP1/TIMP1 ratio(higher in human AD-MSCs) ↓TNF-α, IL-1b and IL-10 in skin(lower in human AD-MSCs) ↓pulmonary fibrosis ↓COL1 and α-SMA transcripts ↓TNF-α ↓IL-1b (lower in human AD-MSCs) NC in IL-10
(Cahill et al. 2016)	Bleomycin intranasal/mice	5 × 104 allogeneic or HGF knockdown BM-MSCs/g bodyweight	intravenous	↓collagen deposition in the lung ↓mRNA expression of IL-1b in the lung ↓protection against fibrosis(treatment with HGF knockdown stem cell) ↓epithelial apoptosis in the lung
(Maria et al. 2016a)	HOCl intradermic injection/mouse (daily, 42 days)	2.5 × 10^5^(the most efficient dose), 5 * 10^5^, or 10^6^ syngeneic BM-MSCs	intravenous	↓skin thickness ↓total collagen content in the skin ↓of COL1, COL3, TGFβ1, and α-SMA in skin ↓Col3 and TGFβ1 in the lung(in a single dose injection on day21) ↓deposition of collagen in lung ↓less ECM deposition ↓cellular infiltration ↓serum AOPP production ↑serum antioxidant capacity ↓anti–Scl-70 antibody serum levels

*Scl-cGVHD* Sclerodermatous chronic Graft Versus Host Disease, *COL1A2* Collagen type I alpha 2 chain, *miR* Micro RNA, *CAV-1* Caveolin-1, *OSM* Oncostatin M, *BAL* Bronchoalveolar lavage, *Trx-1* Thioredoxin 1, *FSP-1* Fibroblast-specific protein 1, *HMGA* High mobility group A, *sRANKL* Soluble Receptor Activator of Nuclear Factor, *CFU-F* Colony-Forming Unit–Fibroblastic, *OCN* Osteocalcin, *ALP* Alkaline phosphatase, *Runx2* Runt-related transcription factor 2, *LPL* Lipoprotein Lipase, *PPARγ* Peroxisome Proliferator-Activated Receptor γ, *TIMP1* Tissue Inhibitor of Metalloproteinase 1, *MMP* Matrix Metalloproteinases, *α-SMA* Alpha-Smooth Muscle Actin, *AD* Adipose, *TNF-α* Tumour Necrosis Factor Alpha, *HGF* Hepatocyte Growth Factor, *BM* Bone Marrow, *IL* Interleukin, *HOCl* Hypochlorous Acid, *MSC* Mesenchymal Stem Cell, *COL* Collagen, *TGFβ* Transforming Growth Factor beta, *ECM* Extracellular Matrix, *AOPP* Advanced Oxidation Protein Products, *NC* No Change

## Clinical studies on mesenchymal stem cells-based treatments for scleroderma subjects

Clinical experiments of MSC-based treatments of SSc are provided in two categories of case reports and clinical trials. AD-MSC, UC-MSC, adipose tissue-derived stromal vascular fraction (ADSVF) and autologous fat are cells/tissues used in the studies we reviewed in order to counteract SSc symptoms. In some cases, regenerative materials have been utilized in combination with platelet-rich plasma (PRP). The reason for adding PRP is its nutritious plasma component that increases the survival of fat graft as well as improving the proliferation of pre-adipocytes through the secretion of particular types of growth factors and cytokines (Daumas et al. 2020). Studies have shown that fat tissue transplantation causes increased skin trophicity leading to better states of mouth opening and digital ulcer healing (Daumas et al. 2020; Del Papa et al. 2019; Onesti et al. 2016). An increased number of capillaries in affected sites were also reported in fat transplantation studies (Del Papa et al. 2019). The beneficial properties of fat tissue that make it suitable for cases affected by skin sclerosis are its biocompatibility, filling property and regenerative potential (due to its high content of multipotent stem or progenitor cells) (Rozier et al. 2018). AD-MSC injection has the same effect on mouth opening range as well as improving hand function and decreasing Raynaud’s sign when applied subcutaneously to all fingers (Onesti et al. 2016; Khanna et al. 2018). ADSVF-based treatments show similar outcomes in enhancing angiogenesis and wound healing causing better hand function and preventing amputation due to hand gangrene (Song et al. 2017; Daumas et al. 2017; Virzì et al. 2017). Other studies have discussed the therapeutic function of UC-MSC in elevating hand and lung function as well as reducing collagen content in the skin in SSc patients (Liang et al. 2018; Wehbe et al. 2016; Zhang et al. 2017). The clinical results and experimental conditions of clinical studies with a publication date of 2016 till now are provided in more detail in Table 4.

**Table 4:** Some of MSC-based Clinical Data For Scleroderma

Source of cells or tissues	References	Type of the study	The site of stem cell injection	Included patients	Clinical outcomes	Post-intervention complications
Fat tissue auto graft ± PRP	(Daumas et al. 2020)	Case report	-PRP/microfat in nasolabial folds and chin and cheekbones -PRP/emulsified fat in upper lip, lower lip, and submucosally at the level of the oral commissure	1 patient Affected by systemic sclerosis–related perioral thickening	↑skin trophicity	-minimal Bruising, pain and swelling in donor areas -superficial hematomas in the injection site
	(Del Papa et al. 2019)	Clinical trial	the base of the finger	25/13 patients with IDU(fat/control groups)	↑IDU healing ↓pain ↑the number of capillaries in the affected digit	NR
Fat tissue auto graft/ AD-MSC	(Onesti et al. 2016)	Clinical trial	Subcutaneous peri-oral location	5/5 affected by dcSSc (fat/ AD-MSC groups)	↑subjective wellness of the skin in the perioral areas ↑IvMHISS score ↑mouth opening ↑VAS score *the results were not significantly different between two groups	NR
AD-MSC	(Khanna et al. 2018)	Clinical trial	Subcutaneous in all fingers	48/40With impairment of hand function(diffuse and limited sclerosis cases)(AD-MSC / control groups)	↑CHFS score ↓RCS ↑SHAQ score Improvement in EQ-5D-5L assessment(diffuse subjects) ↑Patient Global Assessment of SSc activity(diffuse subjects)	-Upper respiratory tract infection -Arthralgia -Cellulitis -Pain in extremity -Hypoesthesia The process is reported to be safe
ADSVF ± PRP	(Virzì et al. 2017)	Case report	Subcutaneous in peri-oral and malar area	6 patients affected by dcSSc	↑skin elasticity (improvement of the opening and extension benchmarks of the labial rhyme) ↓longitudinal skin wrinkles of the upper lip more harmonious, less tense, ↑capillary density ↓vascular ectasia ↑neoangiogenesis	NR
	(Song et al. 2017)	Case report	Subcutaneous in metacarpophalangeal of both hands and the amputation stump of the left middle finger, and under a skin necrosis in the right hand.	62 patients affected by dcSSc	There was no need to further amputation because of gangrene, ulcer and impaired wound healing.	NR
	(Daumas et al. 2017)	Clinical trial	All fingers	12 patients with hand disability of at least 20 points using CHFS.	↑SHAQ score ↑CHFS score ↑grip strength ↑pinch strength ↓ RCS ↓DU NC in Mean global disease severity score	NR
UC-MSC	(Zhang et al. 2017)	Clinical trial	NR	14 patients affected by dcSSc	↓skin thickness ↑lung function (in 3 ILD affected patients) ↑ulcer healing ↓Serum anti-Scl70 autoantibody titer ↓TGFβ and VEGF levels NC in IFN-γ, IL-4 or IL-10	-upper respiratory tract infections -diarrhea
	(Liang et al. 2018)	Retrospective cohort	NR	39 patients affected by SSc	mesenchymal stem cell infusion is a safe therapy for patients with autoimmune diseases	The incidence of Hyper acute (fever, headache, palpitation and so on) and acute (hair loss, facial rash and so on) adverse effects, transplantation-related mortality, infection and malignancy are not high. Survival rate in1 year after infusion is almost 70%
	(Wehbe et al. 2016)	Case report	intravenous	2 affected by progressive, refractory scleroderma	↓dyspnea (first subject) Pulmonary hypertension was normalized (first subject) ↓skin contracture (first subject) pericardial effusion was resolved (first subject) ↓joint pain (second subject) ability to exert was normalized (second subject) ↓arthritis (second subject) ↓Raynaud’s phenomenon pain (second subject) ↑mobility and function(both subjects after second injection)	NR

*PRP* Platelet-Rich Plasma, *IDU* Indolent Digital Ulcers, *IvMHISS* Italian version of Mouth Handicap in Systemic Sclerosis Scale, *VAS* Visual Analogue Scale, *AD* Adipose, *EQ-5D-5L* EuroQol-5-Dimensions-5-Level, *dcSSc* Diffuse cutaneous Systemic Sclerosis, *SSc* Systemic Sclerosis, *SHAQ* Scleroderma Health Assessment Questionnaire, *CHFS* Cochin Hand Function Scale, *RCS* Raynaud’s Condition Score, *DU* Digital Ulcer, *ILD* Interstitial Lung Disease, *TGFβ* Transforming Growth Factor beta, *VEGF* Vascular Endothelial Growth Factor, *IL* Interleukin, *MSC* Mesenchymal Stem Cell, *NR* Not Reported, *NC* No Change

## The most suitable type of mesenchymal stem cells for using in clinical trials of scleroderma

As mentioned, several investigations have been established the advantageous impact of the autologous or allogeneic BM-MSCs and AD-MSCs in different SSc preclinical and clinical cases. Moreover, some of the studies were also evaluated the effects of UC-MSCs (Rozier et al. 2018; Cras et al. 2015). On the other hand, based on some reports, although whole MSCs revealed similar surface markers, various MSC populations from different tissues exhibited varied genetic profiles, and following that they can secrete different factors which can lead to various therapeutic features (Pittenger et al. 2019; Wegmeyer et al. 2013; Marquez-Curtis et al. 2015). In general, the most suitable type of MSC for therapeutic application is the one that can be easily isolated and extracted with less invasive methods. In addition, it can demonstrate its basilar capabilities in higher strength compared to its counterparts from other sources. Accordingly, in contrast to the BM and AD-MSCs, UC-MSCs can be achieved in larger numbers without an invasive method. Furthermore, they have a higher proliferation capacity than BM / AD-MSCs (El Omar et al. 2014). However, some other research has shown that UC-MSC is less prone to adipogenic differentiation (Hematti and Keating 2013). While adipogenic differentiation ability can be helpful in order to attenuate some subcutaneous lesions in SSc individuals (Lee et al. 2017). Collectively, given that the majority of studies related to cell therapy in SSc patients are conducted with BM/AD MSCs, it seems that AD-MSCs are ideal candidates for cell-based treatments in SSc. There are several reasons for this claim, including the following: 1- they can be extracted more easily than BM- MSCs. 2- they have more proliferative, anti-fibrotic, and immunomodulatory potency compared to BM-MSCs (Scuderi et al. 2013; Del Papa et al. 2017). On the other hand, according to the bulk of studies accompanied on the choice between autologous and allogeneic MSC transplantation for the treatment of SSc subjects, cells derived from autologous sources are not appropriate due to alternations in some of the characteristics of MSCs in the micro-environmental conditions caused by scleroderma along with increasing their tendency to myofibroblastic differentiate and increase tissue fibrosis (Rozier et al. 2018; Peltzer et al. 2018).

## Conclusion and future perspectives

The extent of the heterogeneity and severity of scleroderma, as well as the rate of progression in the skin and internal organs, are such that the common treatment options are not completely effective and only can help to alleviate a number of symptoms (Shah and Wigley 2013; Khanna 2011). Hereupon, the evaluation of more efficient approaches is demanded. Given the astonishing outcomes of cell-based therapies for different diseases (Goodarzi et al. 2018b; Rahim and Arjmand 2017; Baradaran-Rafii et al. 2020; Larijani et al. 2020; Arjmand et al. 2019), it isn’t unlikely that cell therapies (specifically using MSCs) will also be effective for individuals with scleroderma. Indeed, MSCs as a practicing member of regenerative medicine with angiogenic, anti-fibrotic, anti-inflammatory, and immunomodulatory functions, can affect the various disordering processes of the disease mainly via secreting specific efficient bioactive molecules (Han et al. 2019). In this context, finding out the most effective type of MSCs and of course, the best dose and method of administration can also shorten the distance to recovery through the MSC-based treatment. Herein, appropriate preclinical (in vitro and in vivo) studies for assessing the safety and efficacy of MSCs can be extremely valuable. Accordingly, based on the preclinical studies around the MSCs transplantation in SSc subjects and comparison of genetic profiles of employed MSCs, it seems that AD-MSCs especially those prepared from allogeneic sources can be considered as gold standard MSCs for clinical application. However, more extensive studies are still needed to find cells with the highest quality. Furthermore, examining the different cell culture conditions for a better cell function and using up-to-date techniques in this area can lead to more effective results. Herein, also increasing the information about the cellular and molecular mechanisms of disease can lead to several incredible advances in the diagnosis and treatment. On the other hand, the advanced omics technology (including genomics, transcriptomics, proteomics, and metabolomics) can be applied to recommend new hypotheses and understand particular underlying mechanisms of diseases (Larijani et al. 2019; Arjmand et al. 2017). Additionally, Knowledge-based computational disease modeling as a worthwhile tool that reduces using animal models, can create a fundamental mechanistic view of the disease. In general, the combination of these multidisciplinary advancements promises progress in recognizing the autoimmune disease and developing targeted therapies. On the other hand, the molecular phenotyping of SSc subjects can provide personalized medicine approaches to specific therapeutic interventions (Arjmand et al. 2017; Arjmand and Larijani 2017) which could raise the possibility of a positive treatment impact.

## Abbreviations

SSc: Systemic Sclerosis
MSC: Mesenchymal Stem Cell
AOPPs: Advanced Oxidation Protein Products
HOCl: Hypochlorous Acid
CCR: CC chemokine receptor
NSIP: Nonspecific Interstitial Peumonitis
CAV-1: Caveolin-1
Th: T helper
IL: Interleukin
PKB: Protein Kinase B
HMGA: High mobility group A
sRANKL: soluble Receptor Activator of Nuclear Factor
CFU-F: Colony-Forming Unit–Fibroblastic
OCN: Osteocalcin
ALP: Alkaline phosphatase
Runx2: Runt-related transcription factor 2
LPL: Lipoprotein Lipase
PPARγ: Peroxisome Proliferator-Activated Receptor γ
RGS5: Regulator of G Protein Signaling 5
CFA: Complete Freund’s Adjuvant
ET_A_R: Anti-endothelin receptor Type-A
AT_1_R: Anti-angiotensin Receptor Type − 1
pSmad2: Phospho-Smad2
bFGF: Basic Fibroblast Growth Factor
CTGF: Connective Tissue Growth Factor
FITC: Fuorescein Isothiocyanate
TSP-1: Thrombospondin-1
BAL: Bronchoalveolar lavage
UIP: Usual Interstitial Pneumonia
RNP: Ribonucleoprotein
CCL: C-C Chemokine Ligand
ECM: Extracellular Matrix
Scl-GVHD: Sclerodermatous Graft Versus Host Disease
Scl-cGVHD: Sclerodermatous chronic Graft Versus Host Disease
PAH: Pulmonary Atrial Hypertension
MCTP: Monocrotaline
SU5416: Semaxanib
BM: Bone Marrow
AD: Adipose
UC: Umbilical Cord
PL: Placenta
HLA-DR: Human Leukocyte Antigen-DR
EC: Endothelial Cell
TGFβRII: TGFβ Receptor II
NGFR: Nerve Growth Factor Receptor
NGF: Nerve Growth Factor
EGF: Epidermal growth factor
ROS: Reactive Oxygen Species
Tsk1/+: Tight skin 1
α-SMA: Alpha-Smooth Muscle Actin
NG2: Neuron-Glial Antigen 2
SA-βGal: Senescence-Associated β-Galactosidase
SDF-1: Stromal Derived Factor-1
CXCR4: C-X-C chemokine receptor
HGF: Hepatocyte Growth Factor
Trx-1: Thioredoxin 1
FSP-1: Fibroblast-specific protein 1
mTOR: Mammalian Target Of Rapamycin
miR: Micro RNA
IDU: Indolent Digital Ulcers
DU: Digital Ulcer
PRP: Platelet-Rich Plasma
ADSVF: Adipose Tissue-Derived Stromal Vascular Fraction
dcSSc: Diffuse cutaneous Systemic Sclerosis
ILD: Interstitial Lung Disease
CHFS: Cochin Hand Function Scale
ADRC: Adipose Derived Regenerative Cell
RCS: Raynaud’s Condition Score
SHAQ: Scleroderma Health Assessment Questionnaire
IvMHISS: Italian version of Mouth Handicap in Systemic Sclerosis Scale
VAS: Visual Analogue Scale
NC: No Change
NR: Not Reported
ND: No Data
PDGFb- R: Platelet-Derived Growth Factor Receptors
TGFβ: Transforming Growth Factor beta
COL: Collagen
MMP: Matrix Metalloproteinases
TIMP1: Tissue Inhibitor of Metalloproteinase 1
VEGF: Vascular Endothelial Growth Factor
FGF: Fibroblast Growth Factor
IGF-1: Insulin-like Growth Factor-1
PDGF: Platelet-Derived Growth Factor
PGE-2: Prostaglandin E_2_
IFN-γ: Interferon-Gamma
TNF-α: Tumour Necrosis Factor Alpha
GILZ: Glucocorticoid-Induced Leucine Zipper
IDO: Indolamine-2,3-dioxygenase
iNOS: Inducible NO Synthase
TSG-6: TNF-α-stimulated Gene-6
IL-1RA: Interleukin-1 Receptor Antagonist
HO-1: Heme Oxygenase-1
TNFR: TNF Receptor
GFP: Green Fluorescent
VEGFR: Vascular Endothelial Growth Factor Receptor
vWF: von Willebrand Factor
SOD: Superoxide Dismutase
PBS: Phosphate-Buffered Saline
OSM: Oncostatin M
EQ-5D-5L: EuroQol-5-Dimensions-5-Level
